# Dual expression of immunoreactive estrogen receptor β and p53 is a potential predictor of regional lymph node metastasis and postoperative recurrence in endometrial endometrioid carcinoma

**DOI:** 10.1371/journal.pone.0188641

**Published:** 2017-11-30

**Authors:** Takeshi Obata, Mitsuhiro Nakamura, Yasunari Mizumoto, Takashi Iizuka, Masanori Ono, Jumpei Terakawa, Takiko Daikoku, Hiroshi Fujiwara

**Affiliations:** 1 Department of Obstetrics and Gynecology, Kanazawa University Graduate School of Medical Sciences, Kanazawa, Ishikawa, Japan; 2 Institute for Experimental Animals, Kanazawa University Advanced Science Research Center, Kanazawa, Ishikawa, Japan; University of South Alabama Mitchell Cancer Institute, UNITED STATES

## Abstract

Although histological grade and muscular invasion are related to the malignant behaviors of endometrial endometrioid carcinoma, lymphatic and/or distant metastases are unexpectedly encountered, even in patients in the low-risk group. To re-evaluate additional reliable parameters to predict the risk of progression, we examined the immunohistochemical expression profiles of p53 and estrogen receptor (ER) β proteins. Patients with endometrial endometrioid carcinoma who underwent surgical treatment at our hospital (n = 154) were recruited to this study, and the significance of the relationships between the incidence of regional lymph node metastasis and/or postoperative recurrence and clinical or experimental parameters was evaluated. By multivariate analysis, we found that histological grades, detection of immunoreactive p53 (positive rates more than 10%, p53-stained), and high expression of ERβ (high-ERβ) were independently associated with metastasis and/or recurrence. Among these parameters, the sensitivity and negative predictive values of high-ERβ were very high (up to 100%). In the population with high-ERβ, the positive rates of metastasis and/or recurrence were 61.1% in the p53-stained group and 21.9% in the p53-non-stained (negative) group. Furthermore, the positive rate in the group showing myometrial invasion of more than 1/2 and showing both p53-stained and high-ERβ was 80%. The disease-free survival of patients who were double-positive for p53-stained and high-ERβ was significantly shorter than that in other patients. In summary, our findings showed that increases in ERβ and p53 immunoreactivity were significantly correlated with the incidence of metastasis and/or recurrence in endometrial endometrioid carcinoma, suggesting that double-positivity for p53-stained and high-ERβ may provide a promising clinical indicator to predict the risk of progression.

## Introduction

Endometrial endometrioid cancer can be categorized into type-I and type-II based on clinical, histopathological, and molecular findings [1]. Type-I endometrioid cancer is mainly composed of well/moderately differentiated cells that are thought to develop in an estrogen-dependent manner [2, 3, 4]. Estrogen receptor (ER) α has been reported to play an important role in the development of estrogen-dependent malignant tumors. However, recent studies have proposed that ERβ is also associated with gynecologic malignant tumors [5, 6]. Moreover, researchers also demonstrated that the expression of ERβ in endometrial endometrioid carcinoma is greater than that in the normal endometrium [5, 7] and that a high ERβ/ERα expression ratio is an independent prognostic marker of survival in patients with endometrial endometrioid carcinoma [8]. Additionally, human endometriotic cells extracted from patients with endometriosis exhibit higher expression of ERβ than cells extracted from patients without endometriosis [9]. Enhanced ERβ activity has also been shown to stimulate the progression of endometriosis through a mechanism involving escape of immune surveillance and interaction with the inflammasome, thereby upregulating interleukin-1β in mice [10]. In type-I endometrial endometrioid carcinoma, dysfunction of DNA mismatch repair genes and gene mutations in phosphatase and tensin homolog deleted from chromosome 10 (*PTEN*) and v-Ki-ras2 Kirsten rat sarcoma viral oncogene homolog (*KRAS*) have been shown to be associated with carcinogenesis of the endometrium [11, 12, 13], whereas gene mutations in *TP53* have been reported to be associated with high-level invasive and metastatic properties of cancer cells [14, 15, 16].

Grades of cellular atypia and differentiation are clinically related to the malignant behaviors of type-I endometrial endometrioid carcinoma [17, 18]. The depth of muscular invasion by cancer cells is an additional important prognostic factor that has been used as a clinical criterion to determine the postsurgical stage of endometrial cancers [18, 19]. Accordingly, endometrioid carcinoma with histological grade 1 (G1) or 2 (G2) along with muscular invasion of less than one-half is classified into the low-risk group [20–23]. Although limited surgical approaches may be applied to patients in low-risk groups, lymphatic and/or distant metastases are unexpectedly encountered, resulting in undertreatment. Therefore, additional parameters to more precisely predict the risk of metastasis are required in order to select appropriate operative procedures.

On the basis of this background, we aimed to identify useful parameters by assessing the relationships between ERβ/p53 and clinical disease progression. In this study, we focused on the immunohistochemical expression of ERβ and p53 protein in endometrial endometrioid carcinoma and investigated whether both ERβ and p53 proteins were associated with the incidence of regional lymph node metastasis or postoperative recurrence.

## Materials and methods

### Tissue samples and patients

Tumor tissues were obtained from patients who had undergone hysterectomy as a primary surgical therapy for endometrial cancer at the Department of Obstetrics and Gynecology, Kanazawa University Graduate School of Medical Sciences (Ishikawa, Japan) between January 2008 and December 2014. All patients were examined under a systemic imaging test to check for distant metastases before surgery, and patients who were found to have distant metastases pre-operatively were excluded from this study. Surgical specimens were fixed with 20% formalin and embedded in paraffin. The tissue sections (4 μm) were stained by routine histopathological techniques for diagnosis. Representative tissue sections from each specimen were subjected to immunohistochemical examination. Fresh tumor tissues from each specimen were frozen and stored at -80°C until use in DNA analysis. Neither chemotherapy nor irradiation therapy was performed prior to primary surgery. Patients who were diagnosed with endometrioid carcinoma on postoperative pathological assessment were recruited to this study. The surgical stage was determined in accordance with the International Federation of Gynecology and Obstetrics (FIGO) staging. Standard surgical procedures for endometrial cancers included hysterectomy and bilateral salpingo-oophorectomy with retroperitoneal lymphadenectomy. Patients with cervical involvement underwent radical hysterectomy and bilateral salpingo-oophorectomy with retroperitoneal lymphadenectomy. Combination chemotherapy or irradiation therapy as postoperative adjuvant treatment was performed for all patients except for those with stage IA and histological grade 1 or 2 (G1 or G2) disease. Clinicopathological information and survival data were extracted from the medical records of each patient. Written informed consent was obtained from each patient. This study and the use of human tissue specimens were approved by the Ethics Committee of the Kanazawa University Graduate School of Medicine.

### Immunohistochemistry

Tissue localization of ERβ and p53 protein was immunohistochemically determined by the avidin-biotin-peroxidase complex (VECTASTAIN ABC Kit; Vector Laboratories, Burlingame, CA, USA) method using formalin-fixed and paraffin-embedded sections, as reported previously [24]. Briefly, sections of representative blocks from each patient were deparaffinized in xylene and rehydrated in ethanol, and antigen retrieval was subsequently performed in 0.01 M citrate buffer (pH 6.0). The slides were immersed in 3% hydrogen peroxide for 10 min to block endogenous peroxidase activity and then washed in 0.05 M phosphate-buffered saline (PBS, pH 7.4). The slides were incubated with primary rabbit monoclonal antibodies against p53 protein (clone: SP5; Thermo Fisher Scientific, Runcorn, UK) at a dilution of 1:100 for 30 min at room temperature and mouse monoclonal antibodies against ERβ (clone: PPG5/10; Bio-Rad Laboratories, Hercules, CA, USA) at a dilution of 1:50 overnight at 4°C in a humidified chamber. After washing, the sections were incubated for 30 min with biotin-labeled goat anti-rabbit IgG for p53 and horse anti-mouse IgG for ERβ at room temperature. Consequently, sections were treated with the avidin-biotin complex at room temperature. Sites of peroxidase activity were visualized with diaminobenzidine (Liquid DAB+ Substrate Chromogen System; Dako, Carpinteria, CA, USA), and the sections were then counterstained with hematoxylin.

The expression profile of p53 was evaluated by estimating the proportion of nuclear staining of tumor cells, as described previously [25]. Cases in which nuclear staining was observed in at least 10% of cancer cells were classified as a p53-stained group. Immunohistochemical staining for ERβ was evaluated based on the percentage of nuclear staining and the intensity of staining in tumor cells, as previously reported [26]. Specifically, the cases were grouped into low expression of ERβ protein (low-ERβ, weak nuclear staining intensity or less than 50% of cancer cells with nuclear staining) or high expression of ERβ protein (high-ERβ, moderate/strong nuclear staining intensity and more than 50% of cancer cells with nuclear staining).

### DNA analysis of *TP53* mutations

DNA was extracted from frozen tumor samples for *TP53* mutation screening using a QIAamp DNA Mini Kit (Qiagen, Hilden, Germany) according to the manufacturer’s instructions. Following DNA extraction, the samples were evaluated for mutations in exons 5–8 of the *TP53* gene because almost all *TP53* gene mutations in various cancers have been found in these exons [27]. Isolated genomic DNA was amplified by polymerase chain reaction (PCR) using the primer pairs 5′-TGTTCACTTGTGCCCTGACT-3′ and 5′-CAGCCCTGTCGTCTCTCCAG-3′ for exon 5, 5′-CTGGGGCTGGAGAGACGACA-3′ and 5′-GGAGGGCCACTGACAACCA-3′ for exon 6, 5′-CTCCCCTGCCACA-3′ and 5′-AGGGGTCAGCGGCAAGCAGA-3′ for exon 7, and 5′-GACAAGGGTGGTTGGGAGTAGATG-3′ and 5′-GCAAGGAAAGGTGATAAAAGTGAA-3′ for exon 8 of *TP53*. PCR was performed for 30 cycles at 95°C for 1 min, 58°C for 1 min, and 72°C for 1 min. After amplification, the resulting PCR products were purified with a MinElute Gel Extraction Kit (Qiagen) or QIAquick PCR Purification Kit (Qiagen). The purified PCR products were sequenced and detected using a DNA sequencer (3730xl DNA Analyzer; Thermo Fisher Scientific).

### Statistical analysis

To analyze progressive factors, chi-squared tests were performed for univariate analysis, and regression analysis was performed for multivariate analysis (IBM SPSS Statistics version 23). Disease-free survival among the 4 groups with p53-/ERβ low, p53+/ERβ low, p53-/ERβ high, and p53+/ERβ high expression was analyzed by the Kaplan-Meier method.

## Results

### Clinical and pathological characteristics

The clinicopathological features of all patients are shown in Table 1. A total of 154 endometrial endometrioid carcinoma cases were included in this study. Among them, 128 patients (83.1%) were postmenopausal, 94 were International Federation of Gynecology and Obstetrics (FIGO) stage IA, 29 were FIGO stage IB, 15 were FIGO stage II, 4 were FIGO stage IIIA, and 12 were FIGO stage IIIC. The mean follow-up time of the 154 patients was 53 months. Regional lymph node metastasis and/or postoperative disease recurrence was observed in 29 (18.8%) cases. In particular, 12 (41.4%) of the 29 patients with metastasis and/or recurrence experienced para-aortic lymph node metastases (Table 2). Two (1.3%) of the patients with FIGO stage IIIC and histological grade 3 (G3) disease who showed myometrial invasion of at least 1/2 died of tumor progression. Regional lymph node metastasis and/or postoperative disease recurrence were observed in 8.3% (8/96) of patients with histological grade 1 disease and in 30.6% (11/36) and 45.5% (10/22) of patients with grades 2 and 3 disease, respectively. The pathological data showed that histological grade 2/3, deep myometrial invasion (≥ 1/2), lymph-vascular space invasion (LVSI), detection of immunoreactive p53 (p53-stained), and high-ERβ were significantly correlated with the incidences of regional lymph node metastasis and/or postoperative recurrence by univariate analysis of metastatic factors (Table 3).

**Table 1:** Clinicopathological data on 154 patients with endometrial endometrioid carcinoma.

		No metastasis		Metastasis	
		n = 125		n = 29	
**Age**		60	(24–87)	60	(36–83)
**Parity**		2	(0–4)	2	(0–3)
**BMI**		22.5	(12.9–40.2)	22.5	(15.1–31.5)
**Preoperative serum CA125 level (U/mL)**		17	(2–545)	21	(7–398)
**FIGO stage**					
	IA	87		7	
	IB	23		6	
	II	13		2	
	IIIA	2		2	
	IIIC			12	
**Histological grade**					
	G1	88		8	
	G2	25		11	
	G3	12		10	
**Myometrial invasion**					
	<1/2	96		9	
	≥1/2	29		20	
**LVSI**					
	Negative	75		6	
	Positive	50		23	
					Median (range)

LVSI: lymph-vascular space invasion

**Table 2:** Summary of the 29 cases with regional lymph node metastasis and/or postoperative recurrence of endometrial endometrioid carcinoma.

No.	FIGO stage	Grade	MI	LVSI	Lymph node dissection	Metastatic lymph node in surgery	Postoperative metastasis
1	IA	G1	<1/2	+	Pelvic LN	No	PAN
2	IA	G2	<	+	Pelvic LN	No	Liver, Cardiophrenic LN
3	IA	G2	<	-	Pelvic LN	No	PAN
4	IA	G2	<	-	Pelvic LN	No	PAN, Peritoneum, Omentum
5	IA	G2	<	-	Pelvic LN	No	Pelvic LN
6	IA	G3	<	+	Pelvic LN	No	PAN, Lung
7	IA	G3	<	+	Pelvic LN	No	Lung, Bone, Mediastinal LN
8	IB	G1	≥	+	Pelvic LN	No	Lung
9	IB	G1	≥	+	Pelvic LN	No	PAN
10	IB	G2	≥	+	No	No	PAN
11	IB	G2	≥	+	No	No	Pelvic LN, Lung
12	IB	G2	≥	+	No	No	PAN
13	IB	G3	≥	+	Pelvic LN	No	PAN
14	II	G1	≥	+	Pelvic LN	No	Lung
15	II	G1	≥	-	Pelvic LN	No	Pelvic LN
16	IIIA	G2	≥	+	No	No	PAN
17	IIIA	G3	≥	+	Pelvic LN	No	Pelvic LN
18	IIIC	G1	<	-	Pelvic LN	Pelvic LN	PAN
19	IIIC	G1	≥	+	Pelvic LN	Pelvic LN	No
20	IIIC	G1	≥	+	Pelvic LN	Pelvic LN	PAN
21	IIIC	G2	<	-	Pelvic LN	Pelvic LN	Lung
22	IIIC	G2	≥	+	Pelvic LN	Pelvic LN	No
23	IIIC	G2	≥	+	Pelvic LN-PAN	Pelvic LN	Lung
24	IIIC	G3	≥	+	Pelvic LN-PAN	Pelvic LN	Lung, Peritoneum
25	IIIC	G3	≥	+	Pelvic LN	Pelvic LN	No
26	IIIC	G3	≥	+	Pelvic LN-PAN	Pelvic LN	Peritoneum
27	IIIC	G3	≥	+	Pelvic LN-PAN	Pelvic LN, PAN	Bone, Lung, Peritoneum
28	IIIC	G3	≥	+	Pelvic LN	Pelvic LN	Peritoneum
29	IIIC	G3	≥	+	Pelvic LN	Pelvic LN	No

MI, muscular invasion; LVSI, lymph-vascular space invasion; LN, lymph node; PAN, para-aortic lymph node.

**Table 3:** Univariate and multivariate analysis of factors affecting disease progression in 154 patients with endometrial endometrioid carcinoma.

		Metastasis		Univariate P-value	Multivariate P-value
**Histological grade**					
	**G1**	8/96	(8.3%)		
	**G2**	11/36	(30.6%)	<0.01	
	**G3**	10/22	(45.5%)	<0.01	0.025
					(G1vs.G2+G3)
**Myometrial invasion**					
	**<1/2**	9/105	(8.6%)		
	**≥1/2**	20/49	(40.8%)	<0.01	0.088
**LVSI**					
	**Negative**	6/81	(7.4%)		
	**Positive**	23/73	(31.5%)	<0.01	0.55
**p53**					
	**Negative**	7/102	(6.9%)		
	**Stained**	22/52	(42.3%)	<0.01	0.014
**ERβ**					
	**Low**	0/86	(0%)		
	**High**	29/68	(42.6%)	<0.01	<0.01

LVSI, lymph-vascular space invasion.

We then conducted multivariate analysis and selected three significant independent metastatic factors: histological grade (G1 versus G2+G3), expression of immunoreactive p53 protein (stained versus non-stained (negative)), and expression of immunoreactive ERβ protein (high versus low; Table 3).

### Relationship between immunohistochemical detection of p53 protein and the incidence of regional lymph node metastasis and/or recurrence

Initially, to evaluate the correlation between the significance of accumulating p53 protein and metastatic/recurrent disease, we evaluated the expression of p53 protein in endometrial tumors by immunohistochemistry. The p53-stained was detected in 52 (33.8%) of the 154 patients with endometrial endometrioid carcinoma. The p53-stained was significantly correlated with a high histological grade (G1: 19/96 [19.8%], G2: 18/36 [50.0%], and G3: 15/22 [68.2%]) and the depth of myometrial invasion (myometrial invasion < 1/2: 26/105 [24.8%] and myometrial invasion ≥ 1/2: 26/49 [53.1%]). Patients showing p53-stained had a significantly increased incidence of metastatic and/or recurrent disease than those with negative staining. In particular, among patients with histological grade G1 or G2, the p53-stained was associated with an increased incidence of metastatic and/or recurrent disease (p53 negative: 5/95 [5.3%], p53-stained: 14/37 [37.8%], *p* < 0.01); however, no differences were observed in patients with histological grade G3 disease (p53 negative: 2/7 [28.6%], p53-stained: 8/15 [53.3%], *p* = 0.27).

The sensitivity and specificity of the p53-stained for regional lymph node metastasis and/or postoperative recurrence were 75.9% (22/29) and 76.0% (95/125), respectively, whereas the positive and negative predictive values were 42.3% (22/52) and 93.1% (95/102), respectively (Table 4).

**Table 4:** Association of p53/ERβ expression and muscular invasion with the incidence of regional lymph node metastasis and/or postoperative recurrence in patients with endometrial endometrioid carcinoma.

	Myometrial invasion						
p53 / ERβ	<1/2		≥1/2		Total		
**- / low**	0/61	(0%)	0/9	(0%)	0/70	(0%)	a
**+ / low**	0/10	(0%)	0/6	(0%)	0/16	(0%)	b
**- / high**	3/18	(16.7%)	4/14	(28.6%)	7/32	(21.9%)	b,c
**+ / high**	6/16	(37.5%)	16/20	(80.0%)	22/36	(61.1%)	a,c

a,b and c: p<0.01

### Relationship between *TP53* gene mutations and the incidence of regional lymph node metastasis and/or recurrence

Next, we examined mutations in the *TP53* gene, which can affect the immunohistochemical detection of p53 protein. Among the 52 cases of p53-stained, we could not perform genetic analysis in 12 cases due to a lack of adequate tissue samples. Consequently, genetic analysis by direct sequencing of PCR products after electrophoresis was performed using tissue specimens from 40 endometrioid carcinoma cases with p53-stained.

**Fig 1:**
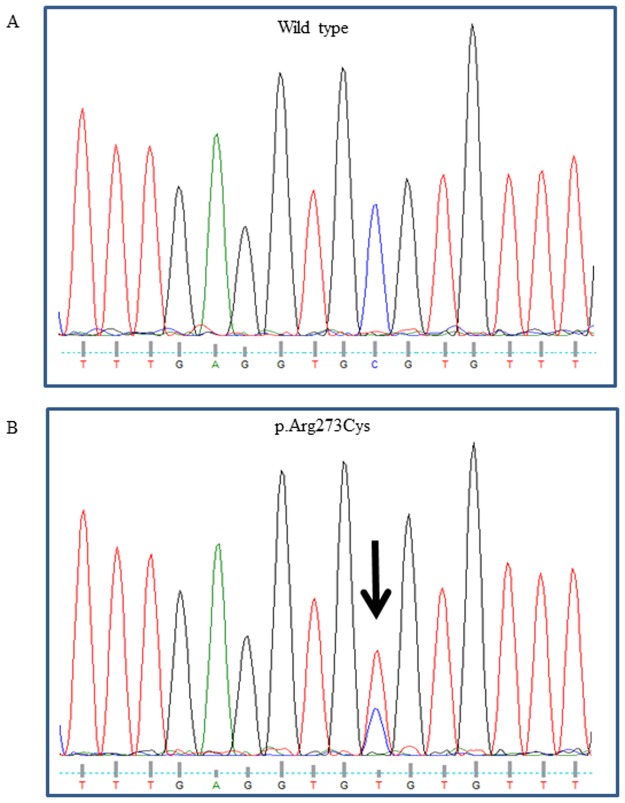
DNA sequence analysis of the *TP53* gene in cases of endometrial endometrioid carcinoma. Representative results of sequencing of exon 8 in the *TP53* gene are shown. The upper panel shows a case of grade 3 endometrioid carcinoma with para-aortic lymph node metastasis and the wild-type sequence for reference (A). The lower panel shows a case of grade 2 endometrioid carcinoma without metastasis and a single nucleotide substitution, cytosine (C) to thymine (T), resulting in an arginine to cysteine substitution at codon 273 of the *TP53* gene (B).

From this analysis, *TP53* gene mutations were observed in 13 (32.5%) of 40 endometrioid carcinoma cases. All of the mutations confirmed by direct sequencing were missense point mutations (Fig 1). Among them, the positive rates were 27.3% in G1 (3/11), 31.3% in G2 (5/16), and 38.5% in G3 (5/13), indicating that *TP53* gene mutations were not significantly correlated with the histological grade in endometrioid carcinoma. In addition, there were no significant differences in the incidences of metastatic and/or recurrent disease between patients with and without *TP53* gene mutation.

Then, we further classified the percent positive ranges of p53 expression into 0–9% (n = 102), 10–19% (n = 22), 20–49% (n = 11), 50–79% (n = 9), and 80–100% (n = 10) and re-evaluated the relationships among p53 expression profiles, p53 mutations, and clinical outcomes (Table 5). High-positive groups (50–79 and 80–100%) showed high rates of p53 mutation (44.4 and 83.3%, respectively), while low-positive groups (10–19 and 20–49%) demonstrated low rates of p53 mutation (16.7 and 14.3%, respectively). On the other hand, the rates of regional lymph node metastasis and/or postoperative recurrence in the low-positive groups (10–19% group (36.4%) plus 20–49% group (45.5%), total (13/33, 39.4%)) were as high as those of the high-positive groups (50–79% group (55.6%) plus 80–100% group (40%), total (9/19, 47.4%)).

**Table 5:** Association of positive ranges of p53 expression, *TP53* gene mutation, and the incidence of regional lymph node metastasis and/or postoperative recurrence in patients with endometrial endometrioid carcinoma.

% of p53 expression	No metastasis			Metastasis				Total mutation	
	Cases	Mutation		Cases		Mutation			
**0–9%**	95	N/A		7	(6.9%)	N/A		N/A	
**10–19%**	14	2/12	(16.7%)	8	(36.4%)	1/6	(16.7%)	3/18	(16.7%)
**20–49%**	6	1/2	(50.0%)	5	(45.5%)	0/5	(0%)	1/7	(14.3%)
**50–79%**	4	3/4	(75.0%)	5	(55.6%)	1/5	(20.0%)	4/9	(44.4%)
**80–100%**	6	3/4	(75.0%)	4	(40.0%)	2/2	(100%)	5/6	(83.3%)
**Total**	125	9/22	(40.9%)	29	(18.8%)	4/18	(22.2%)	13/40	(32.5%)

Incidence of metastatic and/or recurrent disease: 0–9 vs. 10–19%, p<0.01. 10–19 vs. 20–49%, NS. 20–49 vs. 50–79%, NS. 50–79 vs. 80–100%, NS. 10–19 vs. 80–100%, NS.

N/A, not available. NS, not significant.

### Relationship between the expression intensity of immunoreactive ERβ and the incidence of regional lymph node metastasis and/or recurrence

**Fig 2:**
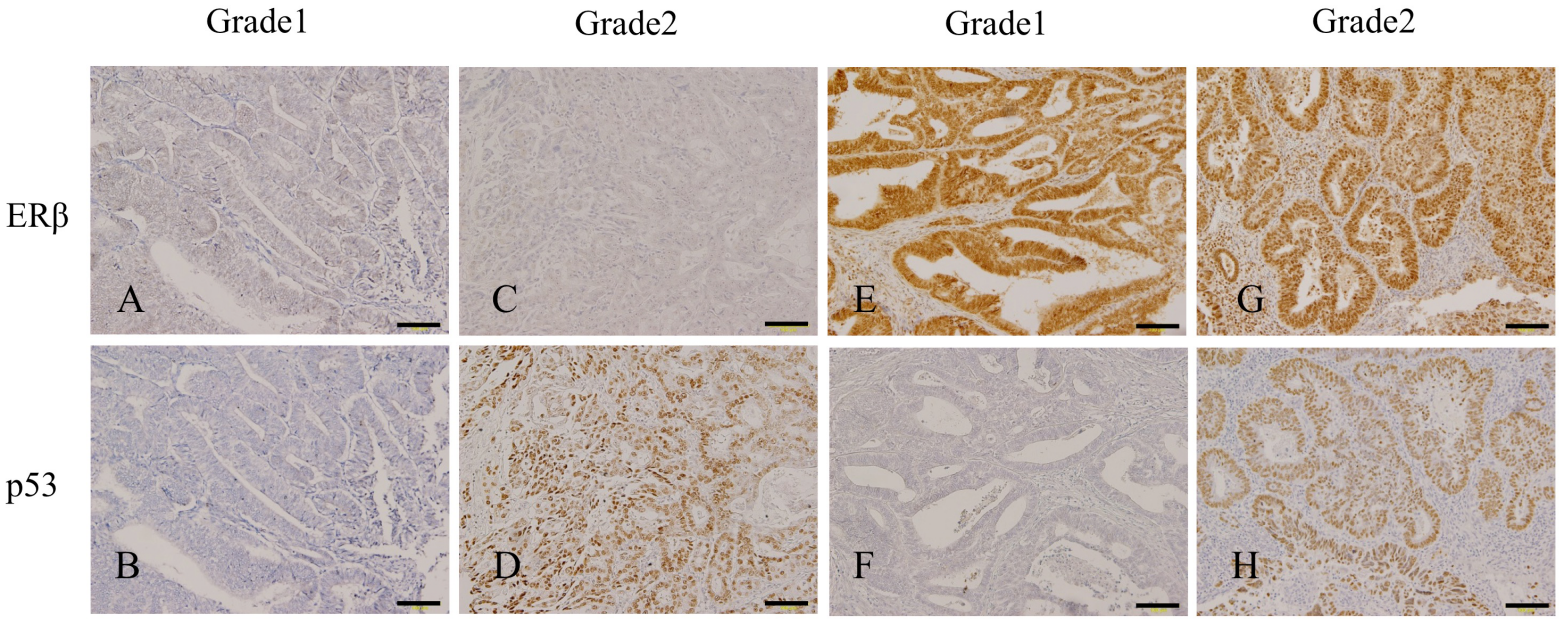
Immunohistochemical localization of ERβ and p53 proteins in representative cases of endometrial endometrioid carcinoma. A and B, Grade 1 endometrial endometrioid carcinoma with low-ERβ (A) and non-stained-p53 (B). C and D, Grade 2 endometrial endometrioid carcinoma with low-ERβ and p53-stained (D). E and F, Grade 1 endometrial endometrioid carcinoma with high-ERβ (E) and non-stained-p53 (F). G and H, Grade 2 endometrial endometrioid carcinoma with high-ERβ (G) and p53-stained (H). Scale bars, 100 μm.

To evaluate the clinical significance of ERβ expression in endometrial endometrioid carcinoma, we examined the immunohistological expression profiles of ERβ protein. Immunohistochemical localization of ERβ and p53 proteins in representative cases of endometrial endometrioid carcinoma is shown in Fig 2. High expression of ERβ was significantly correlated with a high histological grade (G1: 35/96 [36.5%], G2: 16/36 [44.4%], G3: 17/22 [77.3%]) and deep myometrial invasion (myometrial invasion < 1/2: 34/105 [32.4%] and myometrial invasion ≥ 1/2: 34/49 [69.4%]).

The sensitivity and negative predictive values of high expression of ERβ for regional lymph node metastasis and/or postoperative recurrence were both 100% (29/29 and 86/86, respectively), whereas the specificity and positive predictive values were 68.8% (86/125) and 42.6% (29/68), respectively (Table 4).

Among the population with high-ERβ, the positive rates of regional lymph node metastasis and/or postoperative recurrence were 61.1% in the p53-stained group and 21.9% in the p53-non-stained group (Table 4). The positive rate in the p53-stained group was significantly higher than that in the other groups (Table 4). When we added information on the depth of muscular invasion, the positive rate of the group showing myometrial invasion of at least 1/2 and showing both p53-stained and high-ERβ was 80% (Table 4).

**Fig 3:**
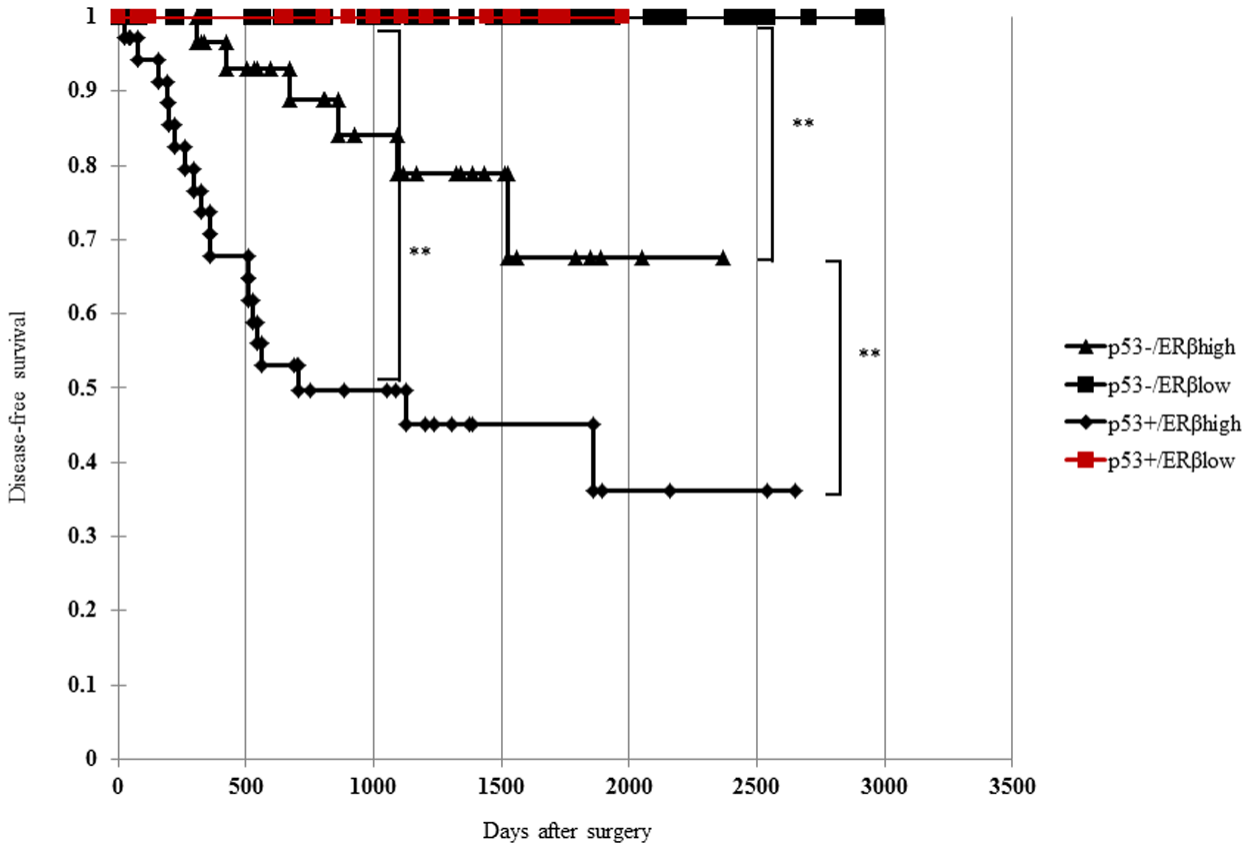
Disease-free survival curve of patients with p53-/ERβlow, p53+/ERβlow, p53-/ERβhigh, or p53+/ERβhigh in endometrial endometrioid carcinoma. Disease-free survival was significantly shorter in patients with p53-stained and high-ERβ (p53+/ERβhigh) than in other patients. Kaplan-Meier survival analysis showed that the disease-free survival of patients with p53-/ERβhigh was significantly shorter than that of patients with p53-/ERβlow and p53+/ERβlow. Patients with p53+/ERβhigh showed significantly shorter disease-free survival than those with p53-/ERβhigh (**: *p* < 0.01).

Kaplan-Meier survival analysis showed that disease-free survival in patients with p53-stained and high-ERβ (p53+/ERβhigh) was significantly shorter than that in other patient groups (Fig 3). The disease-free survival of patients with p53-/ERβhigh was significantly shorter than that of patients with p53-/ERβlow and p53+/ ERβlow. Patients with p53+/ ERβhigh showed significantly shorter disease-free survival than those with p53-/ERβhigh.

## Discussion

In the present study, we showed that expression of immunoreactive ERβ and p53 proteins was associated with the incidence of regional lymph node metastasis and/or postoperative recurrence. To the best of our knowledge, this is the first report to demonstrate a positive correlation between the co-expression of ERβ with p53 protein and clinical outcomes in patients with endometrial endometrioid carcinoma.

Previous reports showed that positive immunostaining for p53 is associated with an increased risk of relapse in patients with low-risk stage I endometrioid endometrial carcinoma [28]. Moreover, p53 mutation is associated with a poor prognosis in patients with endometrial cancer [14, 29]. In accordance with these reports, we found that the positive rate of immunohistochemical staining of p53 protein was significantly higher in patients with regional lymph node metastasis and/or postoperative recurrence than in those without metastatic and/or recurrent disease. To confirm the correlation between the immunohistochemical expression of p53 protein and *TP53* gene mutations, we analyzed the sequences of the established hot-spot areas of the *TP53* gene in patients with p53-stained. However, unexpectedly, our analysis confirmed genetic alterations in *TP53* in only 30% of cases with p53-stained. In addition, there were no significant correlations between *TP53* gene mutations and the incidence of regional lymph node metastasis and/or postoperative recurrence in our cases. Although the detection of accumulated immunoreactive p53 protein in cancer cells by immunohistochemical staining is an established method suggesting the dysfunction of p53 protein [30], our present findings indicated that the observation of positive staining of immunoreactive p53 protein was clinically more effective than the detection of *TP53* gene mutations. Theoretically, the accumulation of p53 protein in cancer cells can be explained by not only gene mutations but also dysregulation of the factors that mediate metabolic or functional cascades involving p53 protein [31]. To support this speculation, when we further classified the p53-stained group into a low-positive group (10–49%) and high-positive group (50–100%), the low-positive group had a low rate of *TP53* gene mutation in spite of a high incidence rate of regional lymph node metastasis and/or postoperative recurrence (Table 5). Considering that the rate of metastasis of the negative group (0–9%) was low (6.9%), these findings suggest that low-positive populations (10–49%) can be classified as a novel high-risk group based on a different perspective from *TP53* gene mutation. Further investigation of these associated factors is necessary to understand the clinical significance of the positive expression of p53 protein in patients with uterine endometrioid carcinoma with poor prognoses.

Importantly, in this study, despite the significance of p53 protein, approximately 25% of cases with regional lymph node metastasis and/or recurrent disease did not express immunoreactive p53 protein. Considering that *TP53* gene mutant tumors also show the null phenotype of immunoreactive p53 expression, it should be noted that the immunohistochemical detection of p53 protein alone may not be sufficient to predict the risk of regional lymph node metastasis and/or postoperative recurrence.

The differentiation of human endometrium is mainly regulated by estrogen and progesterone [32]. Estrogen can act on the endometrium through two main ER isoforms, ERα and ERβ. Although ERβ has been suggested to coordinately play an important role in maintaining the normal function of the endometrium together with ERα, the precise physiological roles of ERβ in endometrial function remain unclear [33]. Similarly, the possible involvement of ERβ in the carcinogenesis of endometrial carcinoma is still being debated [5, 7, 34]. Recently, an abnormal balance between the two ER subtypes was suggested to be involved in carcinogenesis, tumor invasion, and poor clinical outcomes in patients with gynecologic malignant tumors [8, 35]. Additionally, the expression of ERβ protein was reported to be higher in endometrial endometrioid carcinoma than in the normal endometrium [5, 7]. Furthermore, a high ERβ/ERα expression ratio was observed in metastatic lesions and was found to be associated with a poor prognosis [36]. Based on the assessment criteria reported by Konstantinopoulos et al. [26], our study showed that high expression of ERβ was significantly correlated with the incidence of regional lymph node metastasis and/or postoperative recurrence. Notably, more than 80% of patients included in this study developed endometrioid carcinoma after menopause, suggesting that estrogen may not play an important role in the progression of endometrioid carcinoma. Consequently, the possible presence of an estrogen-independent function of ERβ or other intranuclear proteins with similar immunoreactivity as ERβ protein may explain this association between ERβ expression and clinical progression.

Standard operating procedures for the surgical treatment of endometrial cancer include abdominal or laparoscopic hysterectomy and bilateral salpingo-oophorectomy with or without pelvic and para-aortic lymphadenectomy. Because the optimal operating procedure is selected based on pre- and intra-operative evaluations [37–41], determination of suitable clinical parameters to predict the risk of regional lymph node metastasis and/or postoperative recurrence is needed. These parameters are also helpful when determining the appropriate postoperative adjuvant therapy. Although the number of samples is limited, the calculated sensitivity (100%) and negative predictive value of high expression of ERβ for regional lymph node metastasis and/or postoperative recurrence are very promising. In the population with high ERβ expression, the positive rates of regional lymph node metastasis and/or postoperative recurrence were 61.1% in the p53-positive group and 21.9% in the p53-negative group (Table 4). In this regard, double-positive expression of immunoreactive ERβ and p53 proteins may become an excellent clinical parameter to predict future risk since they can be examined prior to surgery using cancer tissue samples obtained through pre-operative endometrial biopsy.

Some reports have described the relationships between ERβ and p53 at the molecular level. Indeed, studies have shown that ectopic expression of ERβ does not change the levels of p53 in MCF10A cells, indicating that ERβ has no direct effect on the expression of p53 in the context of the DNA damage response [42]. In contrast, other studies have reported that mutant p53 is strongly induced in p53-mutated colon cancer SW480 cells, whereas wild-type p53 is strongly downregulated in p53 wild-type colon cancer HCT116 cells in response to ERβ expression [43]. Introduction of ERβ results in retention of p53 in the nucleus and subsequently increases p53 transcriptional activity in p53 wild-type MCF7 cells [44]. Accordingly, the specific mechanisms mediating the functional interactions between ERβ and p53 remain unclear. Further clarification of the molecular relationships between ERβ and p53 could provide a rationale for our finding that the double-positive expression of immunoreactive ERβ and p53 proteins is closely associated with the incidence of regional lymph node metastasis and/or postoperative recurrence.

In conclusion, we showed that increased expression of ERβ and p53 proteins in endometrial endometrioid carcinoma was significantly correlated with the incidences of regional lymph node metastasis and postoperative recurrence. Moreover, we demonstrated that positive staining of immunoreactive p53 protein including the low positive ranges (10–49%) was more effective as a clinical parameter than analysis of *TP53* gene mutations. Although the number of samples was small, the double-positive expression of immunoreactive ERβ and p53 proteins may become a promising clinical parameter to predict the risk of lymph node metastasis and postoperative relapse. Further clarification of the molecular relationship between ERβ and p53 may contribute to clarification of the mechanisms of endometrioid carcinoma progression and yield more useful tools to predict the risk of progression in the future.
